# First Acyclovir Determination Procedure via Electrochemically Activated Screen-Printed Carbon Electrode Coupled with Well-Conductive Base Electrolyte

**DOI:** 10.3390/s24041125

**Published:** 2024-02-08

**Authors:** Katarzyna Tyszczuk-Rotko, Katarzyna Staniec, Damian Gorylewski, Aleksy Keller

**Affiliations:** Institute of Chemical Sciences, Faculty of Chemistry, Maria Curie-Skłodowska University, 20-031 Lublin, Polanddamian.gorylewski@mail.umcs.pl (D.G.);

**Keywords:** acyclovir, screen-printed electrode, well-conductive electrolyte, sensitivity improvement, pharmaceutical samples

## Abstract

In this work, a new voltammetric procedure for acyclovir (ACY) trace-level determination has been described. For this purpose, an electrochemically activated screen-printed carbon electrode (aSPCE) coupled with well-conductive electrolyte (CH_3_COONH_4_, CH_3_COOH and NH_4_Cl) was used for the first time. A commercially available SPCE sensor was electrochemically activated by conducting cyclic voltammetry (CV) scans in 0.1 mol L^−1^ NaOH solution and rinsed with deionized water before a series of measurements were taken. This treatment reduced the charge transfer resistance, increased the electrode active surface area and improved the kinetics of the electron transfer. The activation step and high conductivity of supporting electrolyte significantly improved the sensitivity of the procedure. The newly developed differential-pulse adsorptive stripping voltammetry (DPAdSV) procedure is characterized by having the lowest limit of detection among all voltammetric procedures currently described in the literature (0.12 nmol L^−1^), a wide linear range of the calibration curve (0.5–50.0 and 50.0–1000.0 nmol L^−1^) as well as extremely high sensitivity (90.24 nA nmol L^−1^) and was successfully applied in the determination of acyclovir in commercially available pharmaceuticals.

## 1. Introduction

There are many viruses from the *Herpesviridae* family, known around the world, which infect many living organisms—including humans. These viruses belong to the group of viruses made of double-stranded DNA and are highly specific to the host. There are two types of herpes viruses currently known to attack the human body—Herpes simplex virus type 1 (HSV-1) and type 2 (HSV-2). These viruses differ in terms of the place where the symptoms of infection appear. The herpes virus manifests itself as painful red blisters on various areas of the body, usually on the lips or genitals. Viruses from this family have the ability to cause latent infections. Under the influence of certain stimuli, HSV can be activated and, after an infection, go into a hibernation state. In the case of HSV, the infection is lifelong and there is currently no vaccine against Herpes simplex. In order to shorten the duration of the infection, antiviral drug therapy is often involved. The use of such medicaments slows down the process of skin eruption and reduces contagiousness. According to the literature, 70–90% of the human population are serologically positive for HSV type 2. In addition, some sources have revealed some connections between HSV and Alzheimer’s disease. This means that the problem is global and, to this day, both vaccines and chemical compounds that help treat HSV are being sought. Some of the well-known antiviral drugs which help in the fight against HSV are ganciclovir (GCV), penciclovir (PCV) and acyclovir (ACY)—the last of which will be further discussed [1,2,3,4,5].

Acyclovir (ACY) (specifically 2-amino-9-[(2-hydroxyethoxy)methyl]-3.9-dihydro-6H-purin-6-one) is a guanine analogue that is an antiviral drug. It is valued for its high selectivity and low cytotoxicity, i.e., it is one of the drugs that causes the least harmful side effects. When taken orally, after reaching infected cells, it causes drastic inhibition of DNA replication. Acyclovir is metabolized in the human body by only 15–20%. The rest is excreted in urine in an unchanged form. Therefore, a large amount of this compound ends up in sewage and may threaten living organisms [6,7].

Many methods for the analysis of ACY in various types of samples have been described in the literature, e.g., spectroscopy [8], spectrophotometry [9] and chromatography [10]. To our knowledge, the lowest detection limit (2.22 nmol L^−1^) from these methods was obtained using ultra-high-performance liquid chromatography–heated electrospray ionization–tandem mass spectrometry (UHPLC-HESI-MS/MS) [10]. Among the many instrumental methods, the one with the best price/performance ratio is voltammetry. Voltammetric procedures often allow analysis to be performed directly, without the need for sample preconcentration. Thanks to the high sensitivity and low values of the limit of detection (LOD) and quantification (LOQ) that are possible to obtain, it is feasible to reduce the influence of the sample matrix by dosing smaller amounts of samples into the base electrolyte. Hence, the impact of the interferences on the analytical signal is significantly diminished, which is very useful in the analysis of real samples. It is worth emphasizing that voltammetry is also characterized by low consumption of the sample as well as the reagents, which decrease the cost of a single analysis. Many ACY voltammetric procedures have been presented in the literature. Among them, the lowest limit of detection has been obtained using the sensors and procedures shown in Table 1. The sensors used for their development are commonly expensive and require a lot of effort for sensor preparation.

In this paper, we would like to introduce the first use of the electrochemically activated screen-printed carbon electrode (aSPCE) coupled with a high conductivity of electrolyte (CH_3_COONH_4_, CH_3_COOH and NH_4_Cl) in acyclovir trace-level determination. This electrode consists of a carbon working electrode (WE) with a diameter of 4 mm, a silver reference electrode (REF) and a platinum auxiliary electrode (AUX). To the best of our knowledge, there is no work described in the literature using this type of sensor for ACY analysis. Screen-printed electrodes (SPEs) are widely used for the determination of various types of substances. They also have industrial applications, such as a glucose biosensors for diabetics. SPEs are characterized by a fast response time, simple use and the possibility of mass production (low production cost). Moreover, one of the most important features of these sensors is their disposability, which reduces the impact of contamination during individual analysis. In order to improve the adhesion of graphite to the deposit, epoxy-based polymer binders are used, which improves the mechanical durability of these electrodes. Due to the fact that this binder does not conduct electricity, it inhibits the redox processes taking place at the SPEs [11]. In order to improve their sensitivity, screen-printed electrodes are activated. For this purpose, cyclic voltammetry (CV) scans in 0.1 mol L^−1^ NaOH solution are performed. This treatment causes changes in the surface morphology of the working electrode, increasing the number and size of pores, which is connected with the removal of organic binders used by the manufacturer during electrode preparation [12,13]. After the chemical activation stage, sensors are rinsed with distilled water and are ready for measurements.

Under optimized conditions, differential-pulse adsorption stripping voltammetry (DPAdSV) measurements were performed in a supporting electrolyte (CH_3_COONH_4_, CH_3_COOH and NH_4_Cl) with 0.075 mol L^−1^ concentration and pH = 4.2. Thanks to the application of this solution instead of the well-known acetate buffer, it was possible to significantly increase the analytical signal of ACY. The good conductivity properties of the new (CH_3_COONH_4_, CH_3_COOH and NH_4_Cl) formulation were examined thoroughly and utilized in our previous voltammetric procedures (U(VI), Mo(VI) and V(V) determination) [14,15,16]. The newly developed procedure using DPAdSV and aSPCE is characterized by very good analytical parameters, demonstrating the lowest limit of detection (LOD) among all voltammetric procedures currently described in literature (0.12 nmol L^−1^), a wide linear range of the calibration curve (0.5–50.0 and 50.0–1000.0 nmol L^−1^) as well as extremely high sensitivity (90.24 nA nmol L^−1^), and it was successfully used in the determination of ACY in commercially available pharmaceuticals.

**Table 1:** ACY voltammetric determination procedures with the lowest limit of detection (LOD) value described in the literature.

Technique (Electrode)	Linear Range (nmol L^−1^)	Sensitivity (nA/nmol L^−1^)	LOD (nmol L^−1^)	Analyzed Sample	Ref.
SWAdSV (β-CD/EPPGE)	50.0–600.0 1000.0–9000.0	317.34	7.59	Tablets, urine sample	[17]
DPAASV (Ag NPs/CdS NWs/RG/GCE)	10.0–4000.0 4000.0–40,000.0	-	3.30	Blood serum, tablet and topical cream samples	[18]
SWV (SWNT/Naf/GCE)	10.0–30,000.0	15.40	1.80	Urine sample	[19]
LCAdSV (GCE/TFM)	90.0–530.0	37.00	1.00	Synthetic sample that contains antiretroviral drugs or ATP and DNA	[20]
SWV (γ-Fe_2_O_3_-Bent/CPE)	50.0–800.0	4.22	1.55	Pharmaceutical and urine samples	[21]
DPV (GCE/CNT/ILC/RGO/MnO_2_)	10.0–30,000.0	0.47	0.84	Human serum	[22]
DPAdSV (RuNPs/TBA/PGE)	3.0–30.0 30.0–3000.0	-	0.80	Tablet and urine samples	[23]
SWAdSV (NC/GPE)	0.05–1.0	14.10	0.20	Pharmaceutical and biological samples	[24]
DPAdSV (aSPCE)	0.5–50.0 50.0–1000.0	90.24	0.12	Tablets	This work

Techniques: SWAdSV—square-wave adsorptive stripping voltammetry; DPAASV—differential pulse adsorptive anodic stripping voltammetry; SWV—square-wave voltammetry; LCAdSV—linear cyclic adsorptive stripping voltammetry; SWV—square-wave voltammetry; DPV—differential-pulse voltammetry; DPAdSV—differential-pulse adsorptive stripping voltammetry. Electrodes: β-CD/EPPGE—electropretreated pencil graphite electrode modified with polymerized β-Cyclodextrin; Ag NPs/CdS NWs/RG/GCE—glassy carbon electrode modified with silver nanoparticles/cadmium sulfide nanowires/reduced graphene oxide nanocomposite; SWNT/Naf/GCE—glassy carbon electrode modified with single-walled carbon nanotubes and nafion composite film; GCE/TFM—glassy carbon electrode modified with thin mercury film; γ-Fe_2_O_3_-Bent/CPE—carbon paste electrode modified with nano γ-Fe_2_O_3_ composite and bentonite clay; GCE/CNT/ILC/RGO/MnO_2—_glassy carbon electrode modified with layers of multi-walled carbon nanotubes (CNT), an ionic liquid crystal (ILC), graphene (RGO) and MnO_2_; RuNPs/TBA/PGE—pencil graphite electrode modified by ruthenium nanoparticles and thiobarbituric acid; NC/GPE—nanoclay-modified graphite paste electrode; aSPCE—electrochemically activated screen-printed carbon electrode.

## 2. Materials and Methods

### 2.1. Apparatus

The voltammetric (GPES 4.9) and electrochemical impedance spectroscopic (EIS) (FRA 4.9) measurements were performed on the electrochemical analyzer (µAutolab, Utrecht, The Netherlands, Eco Chemie) using a 10 mL quartz electrochemical cell. For the purpose of the experiments various kinds of screen-printed electrodes (SPEs) with a working electrode diameter of 4 mm (Metrohm, Llanera, Spain) were used. The following sensors were examined: screen-printed carbon electrode SPCE (150) (WE.: C; Aux.: Pt; Ref.: Ag) and SPCE (110) (WE.: C; Aux.: C; Ref.: Ag), as well as commercially available modified electrodes based on SPCE (110) such as carbon nanofiber-modified screen-printed carbon electrodes (SPCE/CNFs), graphene-modified screen-printed carbon electrodes (SPCE/GPH), single-walled carbon nanotube-modified screen-printed carbon electrodes (SPCE/SWCNTs) as well as multi-walled carbon nanotube-modified screen-printed carbon electrodes (SPCE/MWCNTs).

An agate mortar, 0.22 μm Millipore filter, ultrasonic bath (InterSonic, model IS-2, Olsztyn, Poland) and analytical balance (RADWAG, Radom, Poland) were used for standards and sample preparation.

### 2.2. SPCE Activation Procedure

Each series of measurements (after replacing the basic electrolyte with a new one) were started with SPCE (150) electrochemical activation. This process involved 5 cyclic voltammetry scans between 0 and 2 V (scan rate = 100 mV s^−1^) in a 0.1 mol L^−1^ solution of NaOH. After this stage, the obtained aSPCE sensor was thoroughly rinsed with deionized water ready for a new analysis.

### 2.3. Supporting Electrolyte Composition and Other Reagents

Measurements were performed at varying concentrations and pH of phosphate-buffered saline (PBS) (Merck; Darmstadt, Germany), acetate buffer (CH_3_COOH/CH_3_COONa) made by mixing appropriate amounts of CH_3_COONa and CH_3_COOH reagents (Merck; Darmstadt, Germany) as well as buffer solution of CH_3_COONH_4_, CH_3_COOH and NH_4_Cl (prepared by adding the calculated amounts of deionized water purified with Milli-Q system (>18 MΩ cm, Millipore; UK), HCl (30% reagent of Fluka; Charlotte, NC, USA) and CH_3_COONH_4_ (5 mol L^−1^ CH_3_COONH_4_, Sigma-Aldrich; Saint Louis, MO, USA).

Electrode activation solution was made by mixing the appropriate amount of NaOH (Merck; Darmstadt, Germany) with deionized water. Tablets containing acyclovir (200 mg per tablet) from two different manufacturers were purchased at a local pharmacy. Sigma-Aldrich (St. Louis, MO, USA) MS-grade nitric acid appropriately diluted with distilled water was used for sample preparation.

### 2.4. Acyclovir and Interferent Standards Preparation

All acyclovir standard solutions (1 mmol L^−1^, 0.1 mmol L^−1^ as well as 0.01 mmol L^−1^) were prepared by dissolving ACY white powder (Sigma-Aldrich; St. Louis, MO, USA) in deionized water stored in a refrigerator and sonicated in an ultrasonic bath daily for a couple of minutes before use. The 1 mmol L^−1^ solution was prepared once every 3 weeks, in contrast to the 0.1 and 0.01 mol L^−1^ standards which were unstable in the long term. To avoid further complications, these solutions were prepared daily.

The influence of ascorbic acid, adenine, uric acid, Triton X-100 as well as various cationic (Pb^2+^, Zn^2+^, Ni^2+^, Cu^2+^, Cd^2+^, Mg^2+^, Ca^2+^, Sb^3+^, Fe^3+^) and anionic (Cl^−^, NO_3_^−^, NO_2_^−^) interferents on the ACY analytical signal was examined. All reagents were obtained from Sigma-Aldrich (St. Louis, MO, USA).

### 2.5. Pharmaceuticals Preparation

Tablets containing ACY from two different manufacturers were prepared in the same way. Three tablets were weighed and mortared homogeneously. Next, the mass corresponding to the average mass of the three previously weighed tablets was quantitatively transferred into 200 mL volumetric flask and filled with 0.1 mol L^−1^ HNO_3_ solution to the mark. Then, a volumetric flask was placed in an ultrasonic bath for one hour. Finally, the obtained extract was filtered with a 0.22 μm Millipore filter.

### 2.6. Acyclovir DPAdSV Analysis

Differential-pulse adsorptive stripping voltammetry (DPAdSV), aSPCE and the CH_3_COONH_4_, CH_3_COOH and NH_4_Cl solution were used for ACY determination under optimized conditions. Firstly, an electrochemical cleaning step was involved by applying a potential of 1.4 V for 5 s onto the working electrode, while stirring the solution. Next, ACY was accumulated on the aSPCE by stirring the base electrolyte for 60 s (t_acc._) and applying a potential of −0.1 V (E_acc._). Finally, the DPAdSV voltammogram was registered from 0.5 to 1.4 V with the following technique parameters: amplitude (∆E_A_) of 150 mV, a scan rate (ν) of 250 mV s^−1^, modulation time (t_m_) of 6 ms and 5 ms equilibrium time. The baseline was corrected for each voltammogram and the background was subtracted as well.

## 3. Results

### 3.1. Selection of the Sensor and Influence of Electrochemical Activation on ACY Signal

**Figure 1:**
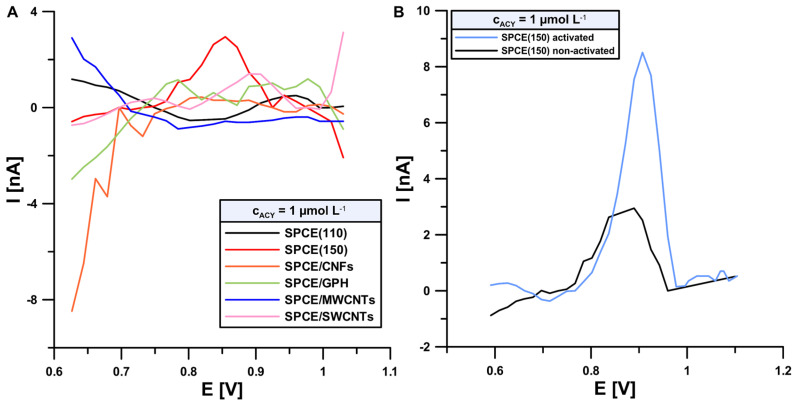
DPV voltammograms registered in 0.075 mol L^−1^ PBS pH = 6.8 in the presence of 1 μmol L^−1^ ACY at (**A**) SPCE (110), SPCE (150), (SPCE/CNFs), (SPCE/GPH), (SPCE/MWCNTs) and (SPCE/SWCNTs); (**B**) the activated and non-activated SPCE (150).

At the first stage of our research, an optimal sensor was established. For this purpose, differential-pulse voltammograms (DPV) were recorded in the presence of increasing concentrations of ACY (1, 3 and 5 μmol L^−1^) under initial conditions (0.075 mol L^−1^ PBS pH = 6.8, DPV parameters: ∆E_A_ of 125 mV, ν of 175 mV s^−1^ and t_m_ of 10 ms) at the following electrodes: SPCE (110), SPCE (150), (SPCE/CNFs), (SPCE/GPH), (SPCE/MWCNTs) and (SPCE/SWCNTs). Figure 1A shows a comparison of the signals registered at these electrodes (1 μmol L^−1^ of ACY). Measurements taken at SPCE/MWCNTs indicate no signal increments corresponding with standard additions. The current signals of ACY obtained with SPCE/CNFs and SPCE/GPH are at the noise level. However, in the case of the other sensors, a 1 μmol L^−1^ acyclovir signal was visible at the following potentials and peak current intensity: 0.94 V, 1.1 nA SPCE (110); 0.85 V, 2.9 nA SPCE (150) and 0.90 V, 1.38 nA (SPCE/SWCNTs). The highest signal (2.9 nA) was obtained at the SPCE (150); therefore, this sensor was chosen for further experiments.

In order to improve the sensitivity, SPCE (150) was electrochemically activated in a strongly alkaline medium (0.1 mol L^−1^ NaOH) in accordance with the procedure already described in our previous studies [12,13]. In the article [13], the changes in the surface morphology and electrochemical properties of the SPCE (150) before and after electrochemical activation (pre-anodization) were examined using scanning electron microscopy (SEM), electrochemical impedance spectroscopy (EIS) and cyclic voltammetry (CV). The obtained results showed the numerous advantages of subjecting the SPCE to the activation process, including a significant increase in the active surface of the working electrode, a decrease in the charge-transfer resistance and improvement of the kinetics of electron transfer. In addition, SEM imaging showed that the electrode gained a highly porous structure as a result of electrochemical activation in a strongly alkaline medium. These factors have a direct influence on the acyclovir signal, which is shown in Figure 1B. The acyclovir signal obtained with the activated sensors (blue line) is wider, better shaped and 2.8 times higher in comparison with the signal obtained at the non-activated electrode (black line).

### 3.2. Supporting Electrolyte Composition—Impact of Concentration and pH Value

**Figure 2:**
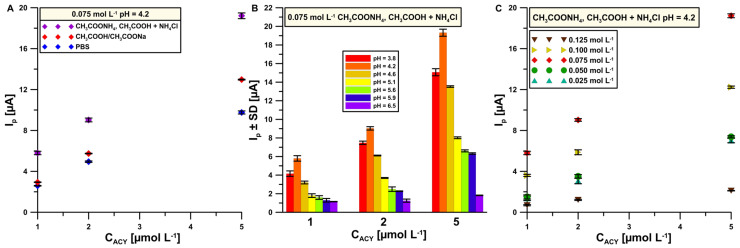
Influence of (**A**) 0.075 mol L^−1^ (pH = 4.2) base electrolyte composition PBS, acetate buffer (CH_3_COOH/CH_3_COONa) and solution of CH_3_COONH_4_, CH_3_COOH as well as NH_4_Cl; (**B**) pH of CH_3_COONH_4_, CH_3_COOH and NH_4_Cl solution; (**C**) concentration of CH_3_COONH_4_, CH_3_COOH and NH_4_Cl solution on I_p_ according to ACY concentration. The standard deviation was calculated for n = 3.

In order to optimize the composition of the basic electrolyte, the measurements were performed in the presence of three ACY additions (1, 3 and 5 μmol L^−1^) in 0.075 mol L^−1^ (pH = 4.2) PBS, acetate buffer (CH_3_COOH/CH_3_COONa) and (CH_3_COONH_4_, CH_3_COOH and NH_4_Cl) solution. The obtained results indicate that the highest peak current intensity (I_p_) of ACY for every acyclovir standard addition is with the new electrolyte composition (CH_3_COONH_4_, CH_3_COOH and NH_4_Cl) (Figure 2A). This is associated with the improvement of the conductivity of the supporting electrolyte, which was confirmed in our previous work [15]. For this reason, the next step relied on examination of the influence of pH (Figure 2B) as well as the concentration of the buffer solution (Figure 2C) on the ACY signal. Finally, a 0.075 mol L^−1^ solution of CH_3_COONH_4_, CH_3_COOH and NH_4_Cl with pH = 4.2 was established as the best choice and used for further studies.

### 3.3. Acyclovir Electrochemical Behavior

**Figure 3:**
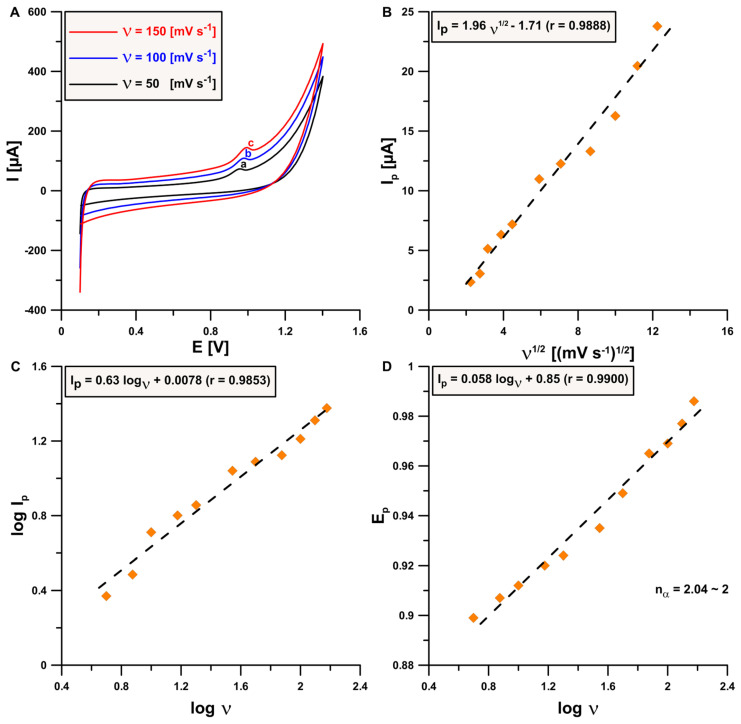
Cyclic voltammetry measurements registered on aSPCE in the presence of 0.1 mmol L^−1^ ACY in optimized electrolyte formulation with a scan rate between 5 and 150 mV s^−1^. (**A**) Selected CV voltammograms; (**B**) dependency between I_p_ and ν^1/2^; (**C**) relationship between log I_p_ and log ν; (**D**) dependency between E_p_ and log υ.

For the next step, a series of cyclic voltammograms at different scan rates (from 5 to 150 mV s^−1^) were recorded in the presence of 0.1 mmol L^−1^ ACY under optimized supporting electrolyte conditions. Increasing the scan rate affects onto the ACY peak potential, which shifts slightly towards more positive values (a → c), as well as peak current intensity (I_p_) values, which increase slightly (Figure 3A). There is no visible reduction peak, which is evidence of the irreversible nature of the examined process. On the basis of relationships between peak current intensity (I_p_) and square root of scanning rate (ν^1/2^) (there is a linear course of that dependency → r = 0.9888) (Figure 3B), as well as log I_p_ and log ν (the slope of the curve is higher than theoretical value = 0.5) (Figure 3C), it was found that the investigated process is mixed (adsorption–diffusion controlled) [25]. In turn, the value of the slope of dependency between peak potential (E_p_) and log υ equal 0.058 was used in the Laviron equation [26] and allows us to conclude that two electrons are involved in the process of electrooxidation of acyclovir at the aSPCE under optimized supporting electrolyte conditions.

**Figure 4:**
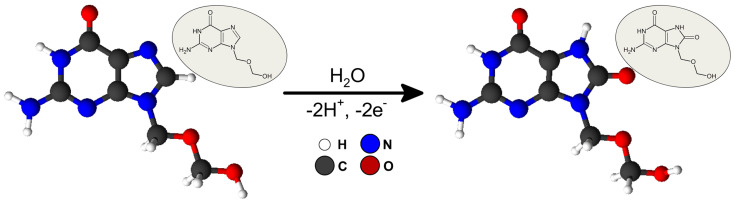
Acyclovir behavior at aSPCE under an optimized basic electrolyte formulation.

According to the data in the literature and the results obtained, the process of acyclovir oxidation relies on deprotonation and cleavage of the double bond in the imidazole ring between the N_(7)_ = C_(8)_ atoms. The described process of ACY electrooxidation into an oxoguanine analogue is shown in Figure 4 [4,27,28].

### 3.4. Optimization of Signal Registration Technique Parameters

**Figure 5:**
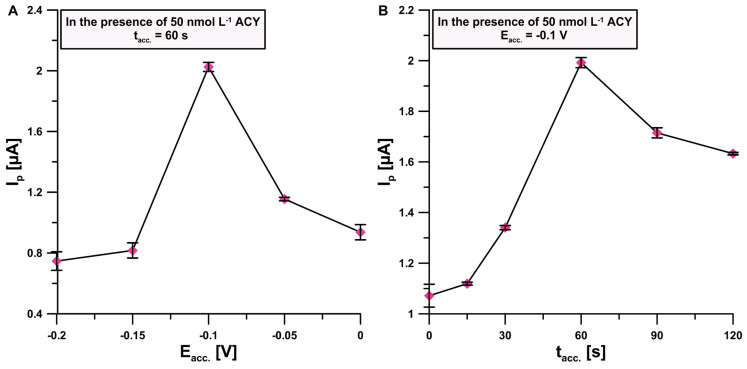
Dependency between (**A**) I_p_ and E_acc._ with a constant value of t_acc._; (**B**) I_p_ and t_acc._ with a constant and optimized value of E_acc._. The standard deviation was calculated for n = 3.

According to our previous conclusions, the process of electrooxidation of acyclovir on aSPCE is mixed. Therefore, the influence of the potential (E_acc._) applied to aSPCE as well as various mixing times (t_acc._) on the ACY analytical signal was examined with the optimized electrolyte composition. Changes in the applied potentials from 0 to −0.2 V with a constant t_acc._ = 60 s value resulted in significant changes in the peak current intensity of the 50 nmol L^−1^ ACY signals (Figure 5A). The best results were obtained for E_acc._ = −0.1 V. For the next step, t_acc_. optimization was performed for constant value E_acc._ and the most effective time was chosen (60 s) (Figure 5B).

**Figure 6:**
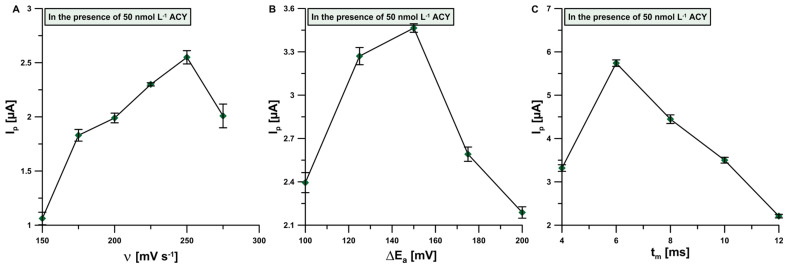
Relationship between I_p_ and (**A**) ν; (**B**) ∆E_A_ as well as (**C**) t_m_. for 50 nmol L^−1^ ACY addition. Experiments were performed in optimized base electrolyte formulation. The standard deviation was calculated for n = 3.

Additionally, the influence of registration technique choice on the 50 nmol L^−1^ ACY signal was examined. The SWV (square wave voltammetry) voltammograms, which we performed with the parameters corresponding to previously used DPV parameters, were very jagged and lacked in the presence of an ACY peak. For this reason, during further optimization, we decided to stick to the DPV technique and optimize the following parameters: scan rate (ν), amplitude (∆E_A_) and modulation time (t_m_). Firstly, the value of ν was changed in the range of 150–300 mV s^−1^ (Figure 6A). At the same time, the values of the potential step and modulation time remained unchanged and were the same as under initial conditions. The ACY peak current increased with the increasing scan rate at a value equal to 250 mV s^−1^ and then started to diminish. Hence, this value was considered optimal. Next, the influence of the ∆E_A_ on the ACY peaks was tested, with the optimized value of ν and constant value of t_m_ (Figure 6B). The best result was obtained with the amplitude value of 150 mV. Finally, the modulation time parameter was optimized in a similar way and t_m_ = 6 ms was selected as optimal (Figure 6C).

For each of the measurements provided, voltammograms were recorded in the range of 0.1 to 1.4 V. However, it was decided to reduce this range, and in subsequent stages of this research, DPAdSVs were recorded from 0.4 to 1.4 V. This change did not affect the peak current intensity but shortened the duration of the analysis and also improved the visibility of the signals.

### 3.5. Analytical Parameters and Robustness Studies

**Figure 7:**
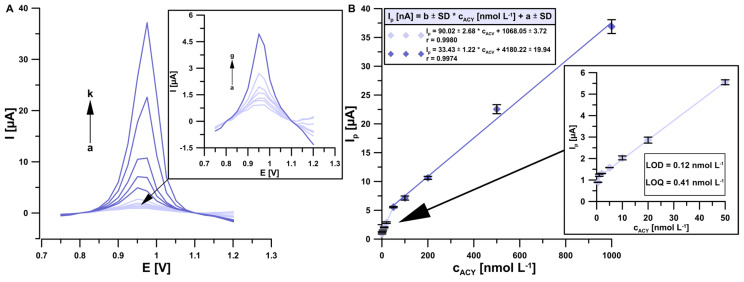
(**A**) DPAdSV voltammograms; (**B**) linear range of calibration curve registered on aSPCE under optimized conditions in the presence of 0.5, 1.0, 2.0, 5.0, 10.0, 20.0, 50.0, 100.0, 200.0, 500.0 and 1000.0 nmol L^−1^ ACY standard additions. The standard deviation was calculated for n = 3.

In the next part of the research, analytical parameters were determined under optimized conditions, such as the calibration curve linear range, the limit of detection (LOD), the limit of quantification (LOQ) and the sensitivity of the developed procedure. DPAdSV voltammograms were registered in the presence of increasing ACY additions a → k (0.5, 1.0, 2.0, 5.0, 10.0, 20.0, 50.0, 100.0, 200.0, 500.0 and 1000.0 nmol L^−1^) (Figure 7A). According to voltammograms, a calibration curve consisting of two linear ranges (0.5–50.0 and 50.0–1000.0 nmol L^−1^) was presented (Figure 7B) and LOD = 0.12 nmol L^−1^ and LOQ = 0.41 nmol L^−1^ values were calculated from the following equations: LOD = 3SD_a_/b and LOQ = 10SD_a_/b, where SD_a_ is the intercept of the standard deviation for n = 3 and b is the slope of the calibration plot [29]. The proposed procedure is characterized by very high repeatability and sensitivity. The RSD values calculated for each of the standard additions were within the range of 0.23–4.90%. Additionally, for the lower range of the linear calibration curve (0.5–50.0 nmol L^−1^), the sensitivity was 90.24 nA/nmol L^−1^, which is one of the best values according to the literature reports presented in Table 1. Moreover, the reproducibility was calculated for the determination of 50 nmol L^−1^ ACY for three sensors. The RSD value was 5.9%, which confirmed the acceptable reproducibility of the aSPCE.

The procedure’s selectivity studies in the presence of interfering species have shown that the 0.1 μmol L^−1^ ACY relative signal was stable and changed by no more than ±10% in the presence of a concentration range of 0.2–2.0 ppm of Triton X-100 and a 5-fold excess of ascorbic acid, adenine, uric acid and cations such as Pb^2+^, Zn^2+^, Ni^2+^, Cu^2+^, Cd^2+^, Mg^2+^, Ca^2+^, Sb^3+^ and Fe^3+^ as well as anions Cl^−^, NO_3_^−^ and NO_2_^−^.

### 3.6. Analysis of Pharmaceuticals

The standard addition method was used for analysis of ACY in tablets intended for cold sore treatment, obtained from two different manufacturers. Table 2 shows results, which indicate agreement between the values declared by both producers. Additionally, the small values for the standard deviation calculated for n = 3 obtained in both determinations prove the high precision of the developed procedure and the recovery indicates a low influence of the sample matrix on the analytical signal in both cases.

## 4. Conclusions

In this paper, a simple and very sensitive voltammetric procedure for trace analysis of acyclovir at an electrochemically activated screen-printed electrode in a good conductive supporting electrolyte has been presented. To the best of our knowledge, this is the first application of a screen-printed electrode in the analysis of this compound described in the literature. Screen-printed electrodes are sensors that are gaining popularity due to their ease of use, cheapness, highly conductive properties as well as good their analytical properties. It has been shown in previous studies that electrochemical activation of SPCE (150), by providing cyclic voltammetry (CV) scans in 0.1 mol L^−1^ NaOH solution, allows for significantly decreased values of charge-transfer resistance and enhances the active surface area of the sensor [13]. Changing the electrochemical properties contributes to the improvement of the quality and intensity of the obtained ACY signals. This electrode, combined with a high conductivity of supporting electrolyte (0.075 mol L^−1^ CH_3_COONH_4_, CH_3_COOH and NH_4_Cl with a pH = 4.2) as well as the DPAdSV technique, allows us to gain a wide linear range of the calibration curve (0.5–50.0 and 50.0–1000.0 nmol L^−1^), extremely high sensitivity (90.24 nA nmol L^−1^) and the lowest limit of detection (0.12 nmol L^−1^) described in literature reports (among voltammetry procedures). The developed procedure enables the determination of acyclovir in pharmaceuticals and, thanks to the use of a screen-printed sensor, it is also possible to perform measurements in field analysis.

## Figures and Tables

**Table 2:** Results of acyclovir determination on the aSPCE in real samples a and b.

Parameter	Sample (Declared Value = 200 mg of Acyclovir per Tablet)
	Tablet a
Found ± SD (n = 3)	187.11 ± 1.95 mg
Recovery	93.56%
Coefficient of variation	1.04%
	Tablet b
Found ± SD (n = 3)	186.90 ± 5.10 mg
Recovery	93.45%
Coefficient of variation	2.73%

Recovery [%] = (found × 100)/declared value; coefficient of variation [%] = (SD × 100)/found.

## Data Availability

The data presented in this study are available on request from the corresponding author.
